# Employing an Immersive Intervention to Remediate Low Mood, Anxiety, and Obsessive–Compulsive Symptoms in Medical Students

**DOI:** 10.1192/bjo.2026.11145

**Published:** 2026-06-30

**Authors:** Alannah Walker, Georgia Davies-Payne, Ludan Tajeldin, Halah Janabi, Athanasios Hassoulas

**Affiliations:** Cardiff University, Cardiff, United Kingdom

## Abstract

**Aims:**

This study evaluated the efficacy of an immersive Acceptance and Commitment Therapy (ACT)-based intervention in reducing subclinical depressive, anxiety, and obsessive–compulsive symptoms in medical students at Cardiff University.

**Methods:**

A quantitative within-subjects repeated-measures design was employed using a single-group pretest–post-test approach. Participants completed baseline measures of obsessive–compulsive (OCI-R), anxiety (GAD-7), and depressive (PHQ-9) symptoms prior to engaging with the immersive ACT intervention, and the same measures were repeated post-intervention. Participants were recruited via the medical school’s student support unit and through advertising across all years of the undergraduate medical programme.

**Results:**

Seventeen participants completed all study components. No significant pre–postdifferences were observed on the total OCI-R, GAD-7, or PHQ-9 scores. However, subscale analyses revealed a significant reduction in the neutralising dimension of the OCI-R following the intervention (z=−1.941, p <0.05). Post-intervention differences were also identified between participants with high versus low depressive symptoms (PHQ-9), specifically in obsessional symptoms (z=−2.298, p <0.05). In addition, participants with high versus low anxiety scores (GAD-7) demonstrated a significant reduction in ordering symptoms post-intervention (z=−2.179, p=0.029).

**Conclusion:**

Although no significant changes were observed on overall measures of obsessive–compulsive, anxiety, or depressive symptoms, targeted reductions were evident in specific symptom dimensions following the immersive ACT-based intervention. These findings suggest potential symptom-specific benefits for medical students with elevated obsessive–compulsive, depressive, and anxiety traits. While limited by a small, subclinical sample, the results support further investigation of immersive ACT interventions in larger and clinical populations.

